# Lecture on Dropsy—Pathological Anatomy of Dropsy, Dependant on Disease of the Heart, and of the Kidneys

**Published:** 1840-02-15

**Authors:** W. W. Gerhard


### CLINICAL LECTURE.

### PHILADELPHIA HOSPITAL.

*Wednesday, February 5th,* 1840.

### LECTURE ON DROPSY—PATHOLOGICAL ANATOMY OF DROPSY, DEPENDANT ON DISEASE OF THE HEART, AND OF THE KIDNEYS.

By W. W. Gerhard, M. D.

*No.* 12—*Winter Course.*

In a demonstrative and somewhat discursive course of lectures, like the present, it is useful occasionally to look back and measure the ground that we have gone over, before we proceed to other subjects of a different nature from those which have principally occupied our attention. During the present course, I have had it in my power to demonstrate to you a great many varieties of pulmonary and cardiac diseases: for such illustrations the season affords the most favourable opportunities. Among the affections which have been presented to your notice, you have witnessed some very rare forms of pulmonary disease, and of their accidental complications. I refer to gangrene of the lungs, and pneumothorax. Two cases of the former have been before the class; of the latter you have seen one case,—and such of you as desire it, have an opportunity of seeing another, which is now in the wards. Of the more frequent forms of pulmonary disease, viz., bronchitis, pneumonia, phthisis, and pleurisy, you have seen numerous examples; and the pathological anatomy of each has been fully demonstrated to you.

You have also had the opportunity of observing many interesting cases of cardiac disease, including hypertrophy, pericarditis, and endocarditis. The knowledge of these affections will be of great importance to you, as, independently of the interest immediately connected with them, they will enable you to understand the nature of many chronic diseases, which cannot be clearly investigated, nor successfully treated, without a proper acquaintance with the primary lesions from which they originate. The season has not offered us many instances of affections of the abdominal viscera. You have nevertheless observed cases of gastritis, intercurrent to other diseases: of dysentery, of jaundice, and of diarrhœa, particularly that form of it occurring in phthisical patients. Cerebral diseases have been more frequent, and in many instances of a grave character. I have presented to you examples of simple acute arachnitis, of tubercular meningitis, and of subacute meningitis,—all of which affections are of frequent occurrence, and often require the attention of the private practitioner. You have also witnessed diseases of the substance of the brain, as softening and apoplexy,—the latter of which was a short time since very fully illustrated by a case of abundant effusion of blood into the corpus striatum and the right ventricle of the brain.

Thus I have illustrated most of the ordinary varieties of disease, with the exception of dropsies and fevers. I shall say something of the former in the present lecture. As to fevers, at the commencement of the course I showed you a few examples of the intermittent and remittent types; but of the two most interesting forms, (typhus and typhoid,) contrary to my expectations, no cases have as yet occurred. But it is probable that I may yet have an opportunity of showing you some cases before the close of the course. For it is unusual for a winter to pass without cases of the latter disease; the former is more irregular in its appearance, and usually occurs only from time to time as an epidemic.

I have, on different occasions, particularly insisted on certain points of great practical importance. One of these is, that certain diseases have a natural course and termination, from which they never deviate, unless some unfavourable circumstance should interfere with the operations of nature. Another of these principles is deduced as a corollary from the preceding: it is, that we should never interfere with such cases of disease by an active treatment, if every thing is going on in the usual favourable manner. This principle is of such immense importance in practice, that I always take the greatest care to urge it upon the attention of the class. A disregard of it has been the source of no little discredit to the medical profession. It is obvious, that when a disease is progressing favourably to a natural termination, all active measures can only annoy the patient, without hastening a successful issue. There are certain mild remedies, which can do no harm; and appropriately used, favour the natural course of things,—and with this view, they may be safely employed. Such are diaphoretics and laxatives. More severe means never fail to distort and aggravate the aspect of the case. But there is a proper medium in this, as in every thing else: it will not do to carry the *expectant* treatment which I have advised, too far. When a positive and manifest benefit can be derived from active measures, our duty to our patients demands that we should employ them. But, I again repeat, that there are many cases in which we can promise ourselves no such good from active treatment, and here our duty equally requires us to abstain from it.

Before proceeding to the proper subject of the lecture, I will show you several patients whom you have lately seen, labouring under acute diseases, and who are now convalescent. The first was before you last week; you will recollect that he was then in the declining stage of pneumonia, which had been attended with severe and protracted hiccough. I then stated that the duration of the case would probably be nineteen or twenty days. The patient began to convalesce on the 27th ult.; the attack commenced on the 8th; so that the disease continued just twenty days. Our treatment, though active, could not diminish this duration; the attack had continued ten days before the patient's entrance; and it is a well settled principle, that no means are competent to cut short an attack of pneumonia, if delayed more than two or three days from its commencement. The disease must go on; all that we can do, is to palliate the symptoms. The hiccough was one of the most troublesome symptoms in this case. This is always an unfavourable circumstance in the prognosis of acute inflammations, because it indicates a considerable degree of disturbance in the nervous system, and because, moreover, it tends to increase the disturbance, by the harrassing motions which it produces. Assafœtida failed to produce any relief in the present instance; but the oil of amber, in doses of six drops, finally quieted the hiccough. Musk would have been used in preference to either of these articles, if there had been any of good quality about the hospital; but the musk which is kept, even in the best drug stores in the city, is often of such bad quality, that it is almost useless as a medicine. The remainder of the treatment in this case was conducted on the ordinary antiphlogistic plan,—by bleeding, cupping, and the administration of opium, calomel, and digitalis.

*Case* 2.—This patient, who has been affected with gangrene of the lungs, you have already seen several times. He has now perfectly recovered from the original disease, but has been seized with bronchitis. This secondary affection arose from our inability to complete the recovery of the patient by the enforcement of those hygienic regulations, which are of so much importance in convalescence from acute disease. The private practitioner derives the most efficient aid from such measures, which we are altogether unable to adopt in hospitals. The patient is also threatened with tubercular disease, the development of which, in a man so far advanced in years, is very uncommon.

*Case* 3.—This is another case of pneumonia; the patient is the old soldier whom you saw at the last lecture. It was a very mild case of the disorder, and readily yielded to the use of gentle diaphoretic means, the principal of which was the infusion of eupatorium.

*Case* 4.—This man had jaundice complicated with slight bronchitis; he is now perfectly well. The jaundice was treated by cups to the right hypochondrium, and saline laxatives. I have lately received from Dr. Jackson of Boston, formerly professor in the medical department of Harvard University, a paper on this disease, in which he lays great stress on the application of leeches in all cases. There can be no doubt that this is an excellent treatment in a great number of cases; whether it is equally efficacious in those which are not inflammatory, remains to be proven. The present case was one of those dependent on slight inflammation of the liver, and yielded readily to cupping and purgation.

*Case* 5.—This man has had a severe attack of pleuro-pneumonia, from which he is now recovered. But he is still very much emaciated, and his cheek is flushed; his emaciation, instead of diminishing with the progress of convalescence, has rather increased. He, therefore, cannot be considered well, and will require the greatest care to prevent the development of a tubercular disease, with which, as I have more than once stated to you, he is threatened. The tendency to this affection was first awakened by an intermittent fever, and was still farther favoured by the supervention of acute pneumonia, before his recovery from the former. Our patient must, therefore, still be considered as in imminent danger. Cases of phthisis, succeeding badly cured intermittents, are by no means unfrequent in districts where miasmatic fevers prevail. There is, perhaps, some foundation for the opinion, that intermittent is a sort of antagonizing disease to phthisis, but this evidently does not hold good, with respect to the enfeebled condition of the body, remaining after attacks of intermittent.

*Case* 6.—We have here a case of genuine acute anasarca. The patient, a man aged thirty-nine, entered the hospital yesterday. He has uniformly enjoyed good health until lately. He has been intemperate during the greater part of his life; but about six months since, he relinquished the use of ardent spirits, finding that he was enfeebled by it. He continued his ordinary occupation until two months since, when he was seized with severe pain in the right knee, which soon began to swell; in a short time, the pain and swelling also attacked the left knee. Frictions with spirits of turpentine and mustard, were used without relief. The swelling gradually extended to the feet, legs, abdomen, and arms. The patient took some doses of salts, and anti-bilious pills; but little or no change was experienced, previously to his entrance into the hospital yesterday. Swelling was then observed in the eyelids, arms, legs, and abdomen; pulse was one hundred, and full; tongue coated; skin warm and dry; legs still painful. The urine was tested both by nitric acid and heat, and found to contain a large quantity of albumen. Cups to the lumbar region, and infusion of senna and salts were ordered.

This is evidently, from the symptoms enumerated, a case of acute dropsy. It is proper that I should fix your attention more particularly on one or two points connected with the case. No swelling was perceptible until two or three months since; it is not, therefore, a dropsy of long standing, which was temporarily relieved and returned. It is very common to witness cases of anasarca, in which the swelling is apparently dissipated by the use of hydragogues and diuretics. But such cases are not cures; the swelling is exceedingly apt to return after a short interval. I recollect a case of this sort, which occurred while I was a resident in an hospital. The dropsy was treated with elaterium by a practitioner of great experience, and, to all appearance, entirely cured; as I saw the patient often, and was more familiar with his case, I requested that the patient should be suffered to remain for a time in the wards; he did so, and in a short time the swelling returned, evidently because the primary affection from which it originated, was not removed by the treatment.

The patient before us, as you perceive, is œdematous over almost the whole body; every part fits on pressure; there is, therefore, a general infiltration of serum into the cellular tissue. The abdomen participates in the general swelling, but no pain is experienced in any part of it. The liver always calls for a very particular examination in dropsy, more especially in ascites. In this case, there is a very slight fulness in the right hypochondrium; but no tenderness on pressure; and the skin is not jaundiced. It is, therefore, pretty evident, that the disease did not originate in the liver. I next examine the spleen, which, in certain diseased states, may give rise to dropsy, as it sometimes does after intermittent fevers; I find that there is no enlargement or pain on pressure. I must, therefore, go to some other organ in which the disorder may be supposed to have commenced. The heart is often concerned in cases of this sort. But our patient has suffered no pain in the region of the heart; nor has he experienced the slightest dyspnœa or palpitations until very lately. We must, therefore, throw the heart also out of the calculation, and transfer our attention to the only other organ which can be considered as the point from which the affection probably originated. This is the kidney. The urine has been tested by nitric acid and heat, and both tests prove that it contains a large quantity of albumen. This kind of urine is known to arise from an organic disease of the kidney, and it is in this organ, therefore, that we must fix the origin of the affection. The disease of the kidneys giving rise to this modification of the urine, has been particularly studied by Bright, Rayer, and others, who have proved that it consists in a preternatural development of the granules of the cortical substance of the kidneys, in consequence of a peculiar inflammation, attended with a deposit of a yellowish substance, whose nature is not perfectly known. This alteration of structure diminishes the healthy secretion of urine, and causes an accumulation of its elements in the blood, which becomes vitiated by the admixture of urea; the superabundant serum is effused into the cellular tissue, while the albuminous portion of the blood is drained from it by the disordered action of the kidneys. The disease thus commencing in the kidneys, is almost always transmitted secondarily to the heart and liver; and *vice versa*, affections of either of these organs, of such a nature as to produce dropsy, in the end, produce a secondary action on the kidneys. We have lately had several cases illustrative of the manner in which disease is thus reflected from one of these organs to the other. In one of these cases, the patient had received an injury of the chest, from lifting a heavy cask, which resulted in inflammation of the heart, and disease of the valves. The obstruction thus opposed to the circulation, produced an anasarcous effusion throughout the body. About three months after the heart became affected, the liver was suddenly attacked by inflammation, which was indicated by jaundice, pain and tension in the right hypochondrium; this was relieved by cupping, and other antiphlogistic measures. After some time, the kidneys exhibited signs of disease. In another case, precisely the same order of phenomena was observed. In a third, the disease commenced, as in the preceding instances, in the heart, and was then communicated to the liver; but the latter organ became implicated only to a slight degree, and by a gradual process; the existence of hepatic disorder was chiefly manifested by a change in the colour of the skin. The kidneys were again last in the chain of diseased action.

In the present case, the kidneys are as yet the only organs obviously affected; the liver offers very slight signs of disease, and the heart still less. But it is very probable that, in the progress of the case, both will become seriously implicated. Even now the action of the heart is spasmodic, and somewhat louder than natural. This change in the action of the heart, is owing to the altered condition of the blood, for you will always find that anemic patients, whose blood is thin and watery, present a quick jerking action of the heart. In the dropsies that supervene after severe attacks of intermittent and remittent fever, the liver is generally first affected, and subsequently the heart and kidneys, at intervals which vary according to circumstances. While I was a resident physician in this hospital, most of the cases of dropsy commenced in this manner, because fevers of this type were then more severe and frequent than they have since been; the consequence was an unusual number of cases of ascites.

I will now show you the evidence of the diseased condition of the kidneys, by exposing the urine to the action of chemical tests. In the earlier stages of the affection, this sign is not a certain one, because the urine may contain too little albumen to be developed by the action of reagents; but in a case so well marked as the present one, there can be no difficulty in the matter. We have unequivocal evidences of the existence of albumen, which, when permanent, as before remarked, is the proof of a specific inflammation of the kidneys, attended with a preternatural development of the cortical granules. In testing with heat, it is necessary that it should be raised to the boiling point of water. A convenient method of applying the test, is to hold a spoon filled with the urine in the flame of a candle. I now exhibit the process to you, and in proportion as the heat rises, you perceive the abundant formation of albuminous floculi. Heat is a better test than nitric acid, because, if the acid be added in excess, it sometimes redissolves the precipitate. You see, however, the very copious precipitate that is produced by the addition of a few drops of nitric acid. In very severe cases, the urine is almost entirely solidified by either of these tests. It is proper that I should caution you against receiving with too implicit confidence, the statements of writers with regard to the frequent occurrence of "Bright's disease" of the kidneys, as the primary affection in dropsies. That it is so, in many cases, there can be no doubt; but it is equally certain that it often succeeds affections of the heart or liver. We should always endeavour to ascertain the real order of events, and not place that first, which is, in fact, only a secondary occurrence.

The value of the albuminous deposit, as a test of the disease of the kidneys, depends upon its permanence; for, in not a few inflammatory disorders, and in certain eruptive fevers, such as scarlatina, the albumen is found; still it is not permanent, but disappears with convalescence. This subject has been studied by Christison and Gregory in Great Britain, and Rayer and Martin-Solon, in France, who have prosecuted the researches of Dr. Bright.

The treatment of the preceding case, has, thus far, been conducted on antiphlogistic principles. Six cups were yesterday applied to each lumbar region, and the infusion of senna and manna ordered. These measures will be repeated from time to time, as may be necessary. Bleeding is only applicable to a limited number of cases; but cupping is always proper, until the inflammation which attends the early stages, at least, is subdued. In addition to antiphlogistic means, we must also adopt measures to procure the evacuation of the dropsical effusion. Diaphoretics and purgatives are the most proper remedies of an evacuant character. Diuretics are generally contraindicated, unless towards the close of the disease, when the inflammation has been subdued; because, being direct stimulants to the kidney, they cannot but aggravate the disease. Sweating and purging are the only safe means. The utility of diaphoretics has been strongly insisted on by Dr. Osborne, who, perhaps, carries his approbation of them too far. Cupping and purgation will, in most cases, answer better than any other treatment.

This variety of dropsy is more frequent in the British islands than in any other part of the world. Whether this is the consequence of the dampness of the climate, or of the free use of certain drinks among the people, is not ascertained; but the former cause is probably the most efficient one. In France, it is certainly less frequent, and so likewise in our own country. Intemperance and cold appear to be the causes which most frequently develope it.

I have already presented to you some cases of that variety of dropsy which originates in disease of the heart. One of these occurred in a mulatto, who lately died in the hospital. He led a most intemperate and dissolute life, during the intervals of time between his discharge and recommitment to various prisons. He was first seized with palpitations and pain in the præcordia. He went to the Dispensary in Fifth street for advice; he was there bled, and directed to take small doses of calomel, digitalis, and Dover's powder. After a short time he discontinued his visits to the Dispensary, and returned to his profligate course of life. He next submitted himself to the treatment of an herb doctor, in whose hands he grew much worse. He then entered the hospital, with the symptoms previously observed, in a greatly aggravated degree. An intense rasping, alternating with a bellows sound, was heard in the first sound of the heart; the second sound was hardly distinguishable; the impulse was immensely increased, and was felt over a much larger space than natural; there was likewise dulness on percussion, dyspnœa, swelling of the face, legs, and abdomen. It was inferred from these signs, that the disease of the heart which resulted in the effusion, consisted in hypertrophy, and obstruction of the semilunar valves of the aorta, accompanied by inflammation of the membranes of the heart.

On examination after death, we discovered the lesions which I now proceed to demonstrate. The internal surface of the left side of the heart presents a fine example of endocarditis. The whole lining membrane is white, thickened, and opaque. On the right fold of the mitral valve is an ulcer a quarter of an inch in diameter; the left fold is likewise ulcerated, and the remaining portions of the valve are injected, thickened, and apparently on the point of ulcerating. The semilunar valves of the aorta are red, thickened, contracted, and stiffened, while the orifice of the aorta is enlarged so much, that you may thrust three fingers into it. The pericardium is roughened and opaque, in consequence of the effusion of lymph; and there is a small band of false membrane proceeding from the aorta to the body of the heart. The inflammation of the pericardium did not supervene until after the entrance of the patient, and was rather of a serous, than of an adhesive kind; and an abundant effusion of serum was likewise found in the cavity of the membrane. The cavity of the left ventricle is dilated to twice the natural size, while the thickness of its parietes is preserved; it is also much hardened. It is, therefore, manifestly a case of hypertrophy, with dilatation, or *eccentric hypertrophy*, as it is termed. The columnæ carneæ are much enlarged, and the tissue of the heart is nearly as firm as that of the uterus in its natural condition.

The right side of the heart presents fewer marks of disease. This is in accordance with the general rule, that inflammation, in a large majority of cases, attacks the left side, and the right side is affected secondarily, if at all. The lining membrane of the right ventricle varies but slightly from the normal condition; the tricuspid valve appears to be slightly inflamed. Hypertrophy affects the right side, almost equally with the left. It is now sufficiently evident that inflammation of the heart, in this case, was the cause of the dropsy, because the signs of it were very clearly marked before the latter supervened.

The liver also offers evidences of disease. It contains rather more blood than it usually does, and the acini are enlarged and hardened. This condition of the acini constitutes the morbid alteration termed *cyrrhosis*, which is certainly very analogous to Bright's disease of the kidneys. The cause of this change in the liver is not well understood. The kidneys are somewhat altered, and exhibit evident marks of the disease of Bright. The granules of the cortical substance are enlarged, and yellow, opaque specks, are visible between the tubuli uriferi.

From this examination, it appears that the dropsical effusion was the result of an affection of the heart; and that after the blood had become altered in its composition in consequence of the effusion, the liver and kidneys were implicated in the morbid process. It is thus that an affection of a single organ will produce a general disease of the system, and this in its turn will give rise to local disorders. The same double play of diseased action is observed in those cases where bronchitis or pneumonia produces disease of the heart; and familiar instances of the same thing might be cited from numerous other affections.

